# Genomic characterization of antimicrobial-resistant *Salmonella enterica* in chicken meat from wet markets in Metro Manila, Philippines

**DOI:** 10.3389/fmicb.2025.1496685

**Published:** 2025-02-13

**Authors:** Michael Joseph M. Nagpala, Jonah Feliza B. Mora, Rance Derrick N. Pavon, Windell L. Rivera

**Affiliations:** ^1^Pathogen-Host-Environment Interactions Research Laboratory, Institute of Biology, College of Science, University of the Philippines Diliman, Quezon City, Philippines; ^2^Microbiology Division, Institute of Biological Sciences, University of the Philippines Los Baños, College, Laguna, Philippines

**Keywords:** *Salmonella enterica*, antimicrobial resistance genes, chicken meat, plasmids, whole genome sequencing, virulence

## Abstract

The emergence of multidrug-resistant (MDR) *Salmonella* is recognized as a significant public health problem worldwide. This study investigated the occurrence of MDR *Salmonella* serovars in chicken meat from wet markets in Metro Manila, Philippines from February to July 2022. Using whole genome sequencing (WGS) and phenotypic antimicrobial resistance (AMR) testing, the serovar, drug resistance, and virulence profiles of *Salmonella* isolates were characterized. Out of 253 chicken cut samples, 95 *S. enterica* isolates representing 15 distinct serovars were recovered. The most common was *S. enterica* serovar Infantis (51.58%), followed by *S.* Brancaster (9.47%), *S.* Anatum (7.37%), *S.* London (7.37%), *S.* Uganda (6.32%), and *S.* Derby (4.21%). Phenotypic AMR testing revealed that 73.68% of the isolates were resistant to at least one drug class, and 45.26% were MDR. A wide array of antimicrobial resistance genes (ARGs) associated with resistance to 12 different drug classes was identified, including three β-lactamase gene variants: *bla*_CTX-M-65_, *bla*_TEM-1_, and *bla*_TEM-176_. Some of these ARGs were located on MDR plasmids, such as those on IncFIB(K)_1_Kpn3, IncFIA(HI1)_1_HI1, and IncX1_1. A total of 131 virulence genes were detected, some of which conferred pESI-like characteristics to *S.* Infantis. These findings highlight a potential public health risk posed by pathogenic MDR *Salmonella* in chicken meat and underscore the urgent need for further research and coordinated AMR surveillance in the Philippines, aiming to stimulate national efforts to combat AMR.

## 1 Introduction

One of the most prominent public health crises worldwide is antimicrobial resistance (AMR), which is the loss of effectiveness of antimicrobials against infections as a result of their abuse and misuse. In 2019, AMR was estimated to have caused 4.95 million deaths worldwide (Murray et al., 2022) which is not far from the 2050 estimate of 10 million deaths annually (O’Neill, 2014). AMR is a multifaceted and gradually spiraling issue that emerged and disseminated through selective pressure from inappropriate human medicine, improper agricultural practices, and environmental pollution. The One Health approach is a multisectoral effort to address AMR and promote human, animal, and environmental health, which are mutually dependent (McEwen and Collignon, 2018). Bacteria exposed to selective pressure from antimicrobials can become resistant and also possess antimicrobial resistance genes (ARGs) that can be mobilized through horizontal gene transfer (HGT) of mobile genetic elements to other potential pathogens, which present significant threats in clinical treatments. This led to the emergence of multidrug resistance (MDR), which is the resistance to three or more groups of antimicrobials (Magiorakos et al., 2012), as well as resistance to last resort antibiotics such as colistin (Algammal et al., 2023). In fact, MDR pathogens have been widely reported in food animals and clinical samples, potentially attributed to the improper usage of this antibiotic in livestock and human medicine (Danaei et al., 2023).

*Salmonella enterica* is one of the most common foodborne pathogens worldwide, comprising over 2,600 serovars. Based on the type of disease, *S. enterica* can be classified as typhoidal or non-typhoidal. Non-typhoidal *Salmonella* (NTS) can cause gastroenteritis and invasive systemic disease (Dieye et al., 2022). NTS is also considered zoonotic, and, therefore, can be carried by many animal species, which can lead to contamination of the environment and food supplies (He et al., 2020; Nhung et al., 2024). Compounding the problem is the ability of NTS to acquire multiple ARGs that confer MDR, limiting treatment options. The World Health Organization (WHO) highlights this issue by categorizing extended-spectrum β-lactamase (ESBL)-producing *Salmonella* and other *Enterobacteriaceae* as critical priorities for research and development of new antimicrobials, while fluoroquinolone-resistant NTS is listed in high priority (World Health Organization, 2024). Therefore, controlling the spread of MDR NTS is crucial for public health, as it impacts food safety throughout the entire farm-to-fork continuum (Nhung et al., 2024).

With the advent of whole genome sequencing (WGS), uncovering important ARGs and virulence factors in foodborne pathogens like *Salmonella* has become a more cost-effective and comprehensive alternative to conventional typing methods for public health surveillance (Yan et al., 2024). This study investigated the AMR and virulence profiles of 95 *S. enterica* isolates from chicken meat sold in wet markets in Metro Manila, employing phenotypic antimicrobial susceptibility testing, WGS, and bioinformatic analysis. While many developed countries have already established their AMR surveillance systems to combat the spread of highly pathogenic MDR pathogens [Global Antimicrobial Resistance and Use Surveillance System (GLASS), n.d.], similar coordinated efforts are lacking in the Philippines. Thus, this study aimed to explore the distribution of ARGs and virulence genes, as well as the serotypes and phylogenetic relationships of MDR *Salmonella* isolates. The findings will provide insights into the current status of AMR in foodborne pathogens in the Philippines, and help inform future public health strategies.

## 2 Materials and methods

### 2.1 Sample collection, isolation, and confirmation of *Salmonella enterica*

Chicken samples, supplied by local farms to slaughterhouses, were collected from public wet markets in Eastern (San Juan City and Quezon City), Northern (Malabon City), and Southern (Muntinlupa City) Metro Manila, Philippines between February and July 2022. Raw cut-up samples, including breast, wings, drumstick, and thigh, were collected in sterile plastic bags and transported to the laboratory for processing. The isolation of *Salmonella* was performed according to the methods outlined by Ng and Rivera (2015). Twenty-five grams of chicken samples were added into 225 mL buffered peptone water (BD Difco, NJ, United States) in a sterile Rollbag^®^ (Interscience, France) and homogenized in BagMixer^®^ 400 (Interscience, France) for 1 min, and incubated at 37°C for 24 h. Following the pre-enrichment, 0.1 mL of the sample was added to 10 mL Rappaport-Vassiliadis (RV) broth (Difco, BD, Sparks, MD) and incubated at 42°C for 24 h. From the incubated RV broth, colony isolation was done on xylose lysine deoxycholate (XLD) agar (BD Diagnostics System, NJ, United States) incubated at 37°C for 18 to 24 h. Presumptive *S. enterica* colonies, i.e., colonies with black centers and clear or transparent halo were then streaked on nutrient agar (BD Diagnostics System, NJ, United States) for PCR confirmation.

Extraction of DNA was done using DNA purification kit (Monarch^®^, New England BioLabs, MA, United States). Confirmation of *S. enterica* isolates was done by amplifying the *invA* gene as outlined by Ng and Rivera (2015).

### 2.2 Antimicrobial resistance testing

The VITEK^®^ 2 Compact 60 ID/AST System and AST-GN70 card panel (bioMérieux, Marcy-l’Étoile, France) were used to test the resistance of the *S. enterica* isolates against 15 antimicrobial agents: Penicillins—ampicillin (AMP); β-lactam combination agent—ampicillin/sulbactam (AMS), and piperacillin/tazobactam (TZP); Cephems—cefazolin (CZN), ceftriaxone (CTR), and cefepime (CEF); Monobactams—aztreonam (AZT); Carbapenems—ertapenem (ETP), and meropenem (MEM); Aminoglycosides—amikacin (AMK), gentamicin (GEN), and tobramycin (TOB); Quinolones—ciprofloxacin (CIP); Glycylcycline—tigecycline (TGC); Nitrofurans—nitrofurantoin (NFN); and Folate pathway antagonists—trimethoprim/sulfamethoxazole (SXT). For quality control, *Escherichia coli* ATCC 25922 was used as the reference strain. For the interpretation of the minimum inhibitory concentration, breakpoints from CLSI M100 34th edition (CLSI, 2024) were used in the analysis.

### 2.3 Whole-genome sequencing, assembly, and bioinformatics analysis

The 95 isolates from chicken cut samples were sent for Illumina library construction and sequencing at the DNA Sequencing Core Facility of the Philippine Genome Center. Libraries were prepared using Nextera XT DNA library preparation kit (ILMN FC-131-1096) following the manufacturer’s protocol. The resulting libraries were checked for size and concentration using TapeStation 2200 and Qubit dsDNA assay. The libraries were sequenced using NovaSeq 6000. The resulting paired-end reads (2 × 150 bp) were checked for quality using FastQC v0.12.1 (Andrews, 2010), and were trimmed using fastp 0.23.2 (Chen, 2023). Unicycler v0.5.0 (Wick et al., 2017) was used as a SPAdes-optimizer in the assembly of the Illumina reads. The quality of the assembled genomes was evaluated using QUAST 5.2.0 (Mikheenko et al., 2018). The assembled genomes (length: 4.6 to 5.7 Mbp; N50: 57.9 Kbp to 754.8 Kbp) were annotated using Prokka 1.14.6 (Seemann, 2014). Serovar prediction was performed using *Salmonella in silico* typing resource (SISTR) tool v1.1.1 (Yoshida et al., 2016). The presence of ARGs, virulence genes, and plasmids were detected using ABRicate V1.0.1 (Seemann, 2020) which combined data with ≥ 95% nucleotide identity and ≥ 60% coverage from CARD-RGI (Alcock et al., 2023), VFDB (Liu et al., 2022), and PlasmidFinder 2.1 (Carattoli et al., 2014; Camacho et al., 2009). In addition, point mutations were screened using AMRFinderPlus (Feldgarden et al., 2021), employing the same nucleotide identity and coverage cutoff. Gene annotations were recovered from the respective databases, unless specified.

### 2.4 Multilocus sequence typing and phylogenetic analysis

The FASTQ raw reads of the 95 *Salmonella* isolates were uploaded to Enterobase (https://enterobase.warwick.ac.uk/). Multilocus sequence typing (MLST) analysis was done using the seven housekeeping gene loci, *aroC, dnaN, hemD, hisD, purE, sucA,* and *thrA*, to identify sequence types (STs) and eBurst Groups (eBGs) of the isolates.

The core-regions of the isolates were analyzed using ParSNP software v2.0.6, with the following *Salmonella* genomes (BioSample No.) included in the analysis: *S.* Typhimurium LT2 (SAMN03470047), *S.* Typhimurium (SAMN10833329), *S.* Saintpaul (SAMN40973940), *S*. Breda (SAMN13906412), *S*. Kentucky (SAMN43547925), *S.* London (SAMN38156060), *S.* Anatum (SAMN41786477), *S.* Isangi (SAMN08951104), *S.* Amager (SAMN44253386), *S.* Uganda (SAMN43080519), *S.* Livingstone (SAMN02698174), *S.* Lexington (SAMN02843465), *S.* Derby (SAMN14341256), *S.* Albany (SAMN43079470), *S.* Brancaster (SAMN10425346), and *S.* Infantis (SAMN44253386). Identical and unique sequences across all genomes were identified to make multiple sequence alignment. From 112,878 core genome single nucleotide polymorphism (SNP) alignment, a maximum likelihood tree was inferred using RAxML-NG v1.2.2 GTRGAMMA substitution model with 100 bootstrap replicates (Kozlov et al., 2024). The tree was visualized, mid-rooted, and annotated using iTOL v 6.9.1 (Letunic and Bork, 2024).

### 2.5 Data visualization

The heatmaps of ARGs, plasmid replicons, and virulence genes were created using TBTools (Chen et al., 2020). In the analysis, the proportion of serovars possessing ARGs, virulence genes, and plasmids was indicated by values between 0 and 1, and default Euclidean distance and complete clustering method were employed.

## 3 Results

### 3.1 Serovars, MLST, and eBGs

**Figure 1:**
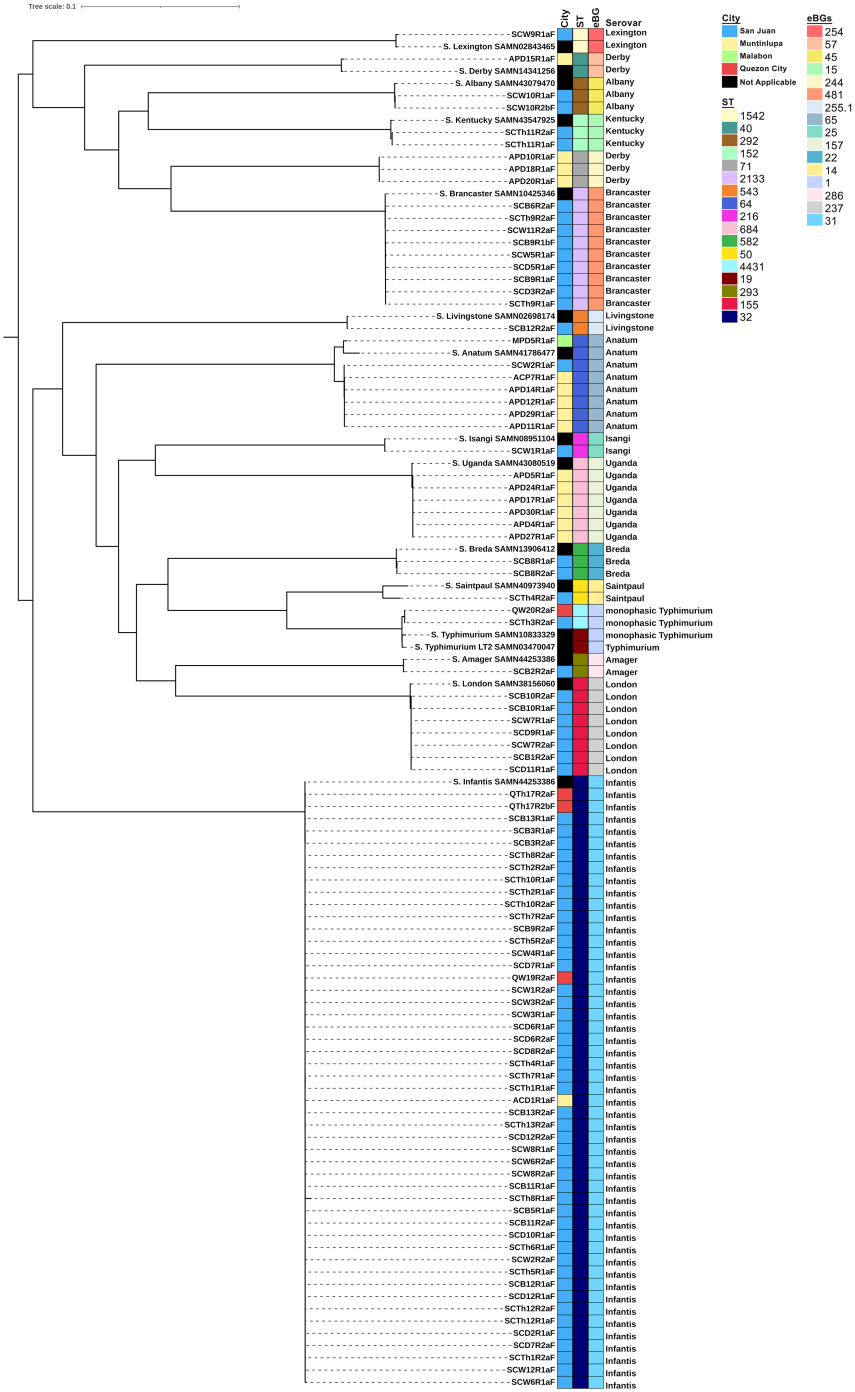
Phylogenetic tree of *S. enterica* isolates (*n =* 95). A core genome analysis was performed using ParSNP software v2.0.6 and a maximum likelihood tree was inferred using RAxML v.1.2.2. The tree was visualized, mid-rooted, and annotated using iTOL v 6.9.1. Sampling location, sequence type (ST), eBurst Groups (eBGs) and serotypes are visualized with the phylogenetic tree. *Salmonella enterica* serovar Typhimurium LT2, BioSample No. SAMN03470047, was used as the reference genome.

Of the 253 chicken cuts collected from four cities in Metro Manila between February and July 2022, 95 isolates of *S. enterica* were recovered: 74 (77.89%) from San Juan City, 17 (17.89%) from Muntinlupa City, 4 (4.21%) from Quezon City, and 1 (1.05%) from Malabon City. *In silico* serotyping using SISTR (Figure 1) revealed that these isolates belonged to 15 distinct serovars. The most frequent serovar was *S.* Infantis, accounting for 51.58% (*n =* 49) of the isolates, followed by *S.* Brancaster (9.47%, *n =* 9), *S.* Anatum (7.37%, *n =* 7), *S.* London (7.37%, *n =* 7), *S.* Uganda (6.32%, *n =* 6), and *S.* Derby (4.21%, *n =* 4). Other serovars included monophasic *S.* Typhimurium I 1,4,[5],12:i:-, *S.* Breda, *S.* Albany, and *S.* Kentucky, each representing 2.11% (*n =* 2). Single isolates of serovars Livingstone, Lexington, Saintpaul, Amager, and Isangi were also identified.

San Juan City yielded 13 distinct serovars, Muntinlupa City had four, Quezon City had two, and Malabon City had one. Monophasic *S.* Typhimurium I 1,4,[5],12:i:-, Anatum, and Infantis were isolated from more than one city, whereas the remaining serovars were isolated from a single location. Based on the MLST, these serovars belonged to 16 distinct sequence types (STs) and eBurst Groups (eBGs), with each serovar assigned a unique ST and eBG, except for *S.* Derby, which was associated with two STs (ST 40 and ST 71) and eBGs (57 and 244). From the maximum likelihood tree, these Derby isolates formed different clades separated by 27,401 SNP differences. Interestingly, these Derby isolates were all isolated from Muntinlupa, with the lone ST 40 possessing more ARGs than the three ST 71. All isolates clustered together with their respective reference genome, except for monophasic *S.* Typhimurium I 1,4,[5],12:i:-, which formed a closely related sub-clade with SAMN10833329 due to 723 SNP differences.

### 3.2 Antimicrobial resistance phenotype

**Figure 2:**
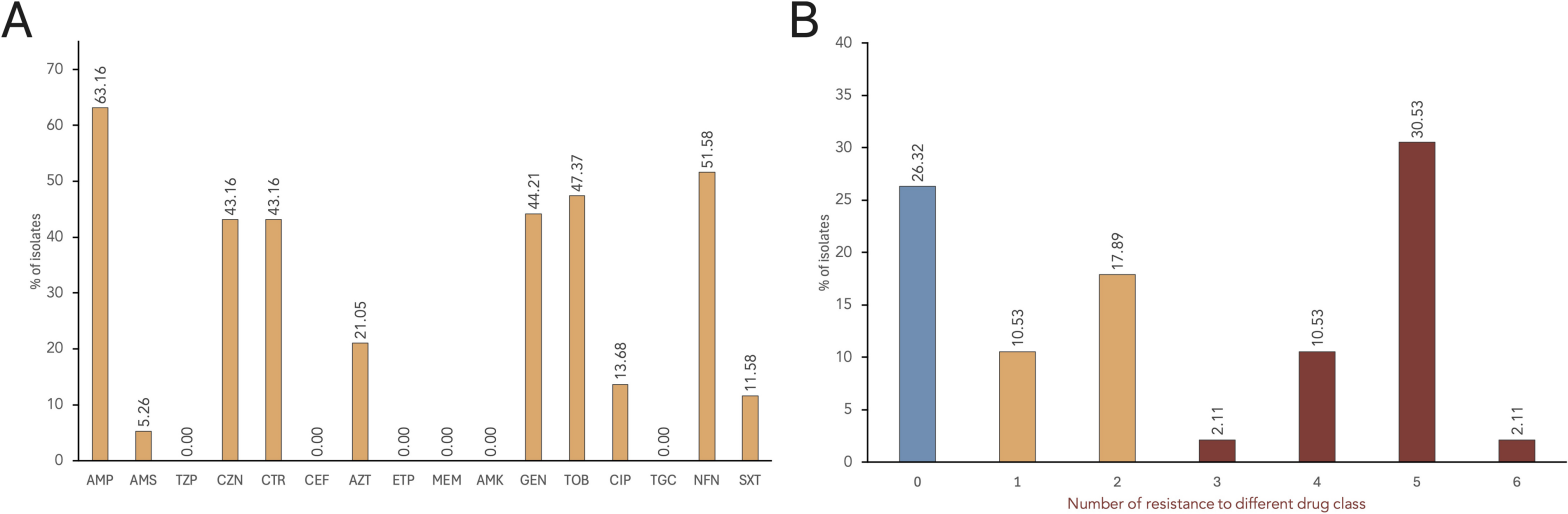
AMR rates of *S. enterica* isolates. **(A)** The proportion of isolates showing resistance phenotype against each antimicrobial tested. Antimicrobials tested: Penicillins—ampicillin (AMP); β-lactam combination agent—ampicillin/sulbactam (AMS), and piperacillin/tazobactam (TZP); Cephems—cefazolin (CZN), ceftriaxone (CTR), and cefepime (CEF); Monobactams—aztreonam (AZT); Carbapenems—ertapenem (ETP), and meropenem (MEM); Aminoglycosides—amikacin (AMK), gentamicin (GEN), and tobramycin (TOB); Quinolones—ciprofloxacin (CIP); Glycylcycline—tigecycline (TGC); Nitrofurans—nitrofurantoin (NFN); and Folate pathway antagonists—trimethoprim/sulfamethoxazole (SXT). **(B)** Distribution of multidrug resistant *S. enterica* isolates.

Based on VITEK^®^ 2 AST results, the most common resistance phenotypes observed among the *S. enterica* isolates were against ampicillin (AMP; 63.2%), nitrofurantoin (NFN; 51.6%), tobramycin (TOB; 47.4%), gentamycin (GEN; 44.2%), cefazolin (CZN; 43.2%), ceftriaxone (CTR; 43.2%), aztreonam (AZT; 21.0%), and ciprofloxacin (CIP; 13.7%) (Figure 2A). Trimethoprim/sulfamethoxazole (SXT) resistance was observed in 11.6% of the isolates, while only five isolates (5.3%) were resistant to ampicillin/sulbactam (AMS). All isolates were susceptible to piperacillin/tazobactam (TZP), cefepime (CEF), ertapenem (ETP), meropenem (MEM), amikacin (AMK), and tigecycline (TGC).

Seventy isolates (73.7%) exhibited resistance to at least one antimicrobial drug class, while 25 isolates were susceptible to all tested antimicrobials (Figure 2B). A total of 17 distinct AMR profiles were identified among the 95 *S. enterica* isolates (Supplementary Table S1). MDR, or resistance to three or more drug class (Magiorakos et al., 2012), was observed in 45.3% of the isolates (*n =* 43), with the majority being *S.* Infantis (*n =* 37). Most of the isolates (30.5%) showed resistance to five drug classes, with AMP-CZN-CTR-AZT-GEN-TOB-NFN as the common MDR phenotype. Furthermore, resistance to six antimicrobial classes was observed in two *S.* Infantis isolates: SCD2R1a (AMP-CZN-CEF-GEN-TOB-CIP-NFN-SXT) and SCTh13R2a (AMP-CZN-CEF-AZT-GEN-TOB-NFN-SXT).

### 3.3 Antimicrobial resistance genes

**Figure 3:**
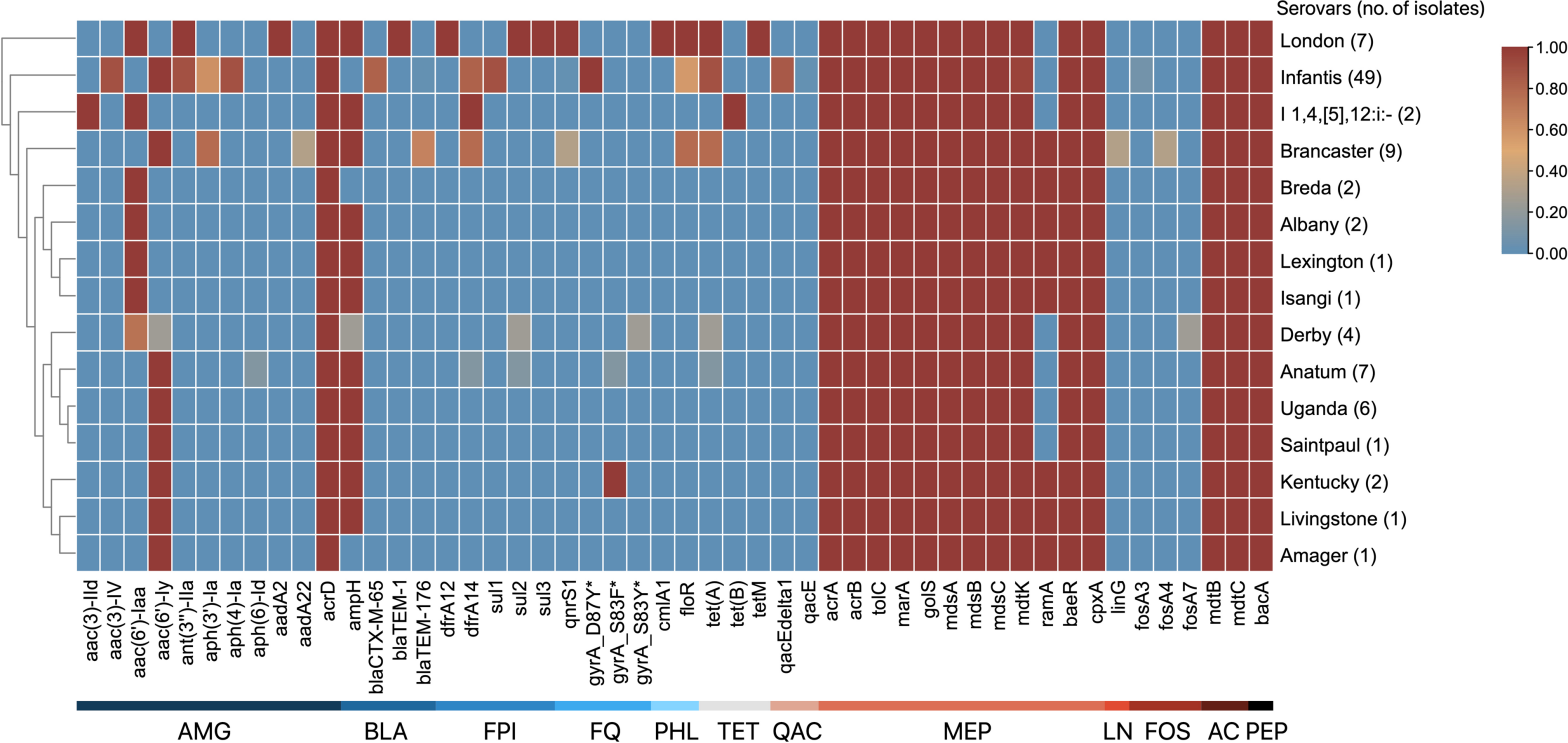
ARG patterns of *S. enterica* serovars. The serovars are clustered by their similarities in ARGs. The antimicrobial class of ARGs are indicated by the labels at the bottom: aminoglycosides (AMG), β-lactams (BLA), folate pathway inhibitors (FPI), fluoroquinolones (FQ), phenicols (PHL), tetracyclines (TET), quaternary ammonium compounds (QAC), multidrug (MEP), lincosamides (LN), fosfomycins (FOS), aminocoumarins (AC), and peptide antibiotics (PEP). The proportion of serovars possessing ARGs ranges from 0 to 1, where absence is 0 and is blue, and presence in all isolates is 1 and is red.

A total of 50 ARGs conferring resistance to 12 distinct drug classes using CARD and AMRFinderPlus were identified (Figure 3). These drug classes include aminoglycosides, β-lactams, folate pathway inhibitors, fluoroquinolones, phenicols, tetracyclines, aminocoumarin, phosphonics, lincosamides, and peptide antibiotics. The identified ARGs are either acquired or produced through point mutations and are encoded in the chromosome or plasmids. Notably, 11 of these genes encode subunits of multidrug efflux pumps that confer resistance to multiple antibiotics. The number of ARGs per genome ranges from 22 to 36 genes, with *S.* London and *S.* Infantis isolates harboring the highest number of ARGs.

Of the 50 ARGs identified, 12 (24.0%) confer resistance to aminoglycosides. The *acrD*, gene encoding an efflux pump, and *kdpE*, a transcriptional regulator, were present in all isolates. In addition, gene variants that encode aminoglycoside modifying enzymes — *aac, aadA*, and *ant—*were identified in several serovars. Four distinct β-lactam resistance genes were identified: *ampH*, which was present in all serovars except *S.* Infantis, *S.* Breda, *S.* Amager, and three *S.* Derby isolates; *bla*_CTX-M-65_ found in 40 of 49 *S.* Infantis isolates; *bla*_TEM-1_, detected in serovar London; and *bla*_TEM-176_, identified in 6 of 9 *S.* Brancaster isolates. Two antifolate resistance genes were observed: *dfrA12*, found in serovar London, and *dfrA14,* which was present in monophasic *S.* Typhimurium isolates, and selected *S.* Brancaster, *S.* Infantis, and *S.* Anatum isolates. Three variants of the *sul* gene, which confers resistance to sulfonamides, were identified in certain serotypes: *sul1* was detected in 44 of 49 of *S.* Infantis isolates; *sul2* was found in all *S.* London, one *S.* Anatum, and one *S.* Derby isolates; and *sul3* was identified in all *S.* London isolates. For quinolone and fluoroquinolone resistance, *qnrS1* was found in all *S.* London, and some *S.* Brancaster, and *S.* Infantis isolates. In addition, point mutations in the DNA gyrase subunit A were also noted: *gyrA_D87Y* detected in all *S.* Infantis isolates; *gyrA_S83F* in *S.* Kentucky and one *S.* Anatum isolate; and *gyrA_S83Y* in one *S.* Derby isolate. Regarding chloramphenicol resistance, only two efflux pump genes were detected among the isolates: *cmlA1* found only in serovar London, and *floR* detected in *S.* London isolates, and selected *S.* Brancaster and *S.* Infantis isolates. For tetracyclines, three resistance genes were identified: *tet(A)* in *S.* London isolates, and selected *S.* Anatum, *S.* Derby, *S.* Brancaster, and *S.* Infantis isolates; *tet(B)* in monophasic *S.* Typhimurium; and *tetM* in *S.* London.

Several multidrug efflux pump component genes were detected across all isolates: *acrAB-tolC* genes, which code for a tripartite resistance-nodulation-division (RND) efflux pump, and their positive regulators *sdiA* and *marA,* that can transport tetracyclines, phenicols, rifamycins, penams, glycylcyclines, cephalosporins, fluoroquinolones, and other disinfecting and antiseptic agents out of the cell*; mdsABC,* which code for another tripartite RND-type efflux pump, and its positive regulator *golS*, used in exporting β-lactams, chloramphenicol, and thiamphenicols; and *baeR* and *cpxA,* which are associated in pumping out aminocoumarin and aminoglycoside antibiotics. Other notable ARGs include gene variants that encode resistance to fosfomycins (*fosA3*, *fosA4*, and *fosA7*); genes *qacE/E∆1* in *S.* Infantis that confer resistance against quaternary ammonium compounds, such as benzalkonium chloride.

### 3.4 Plasmid profiles

**Figure 4:**
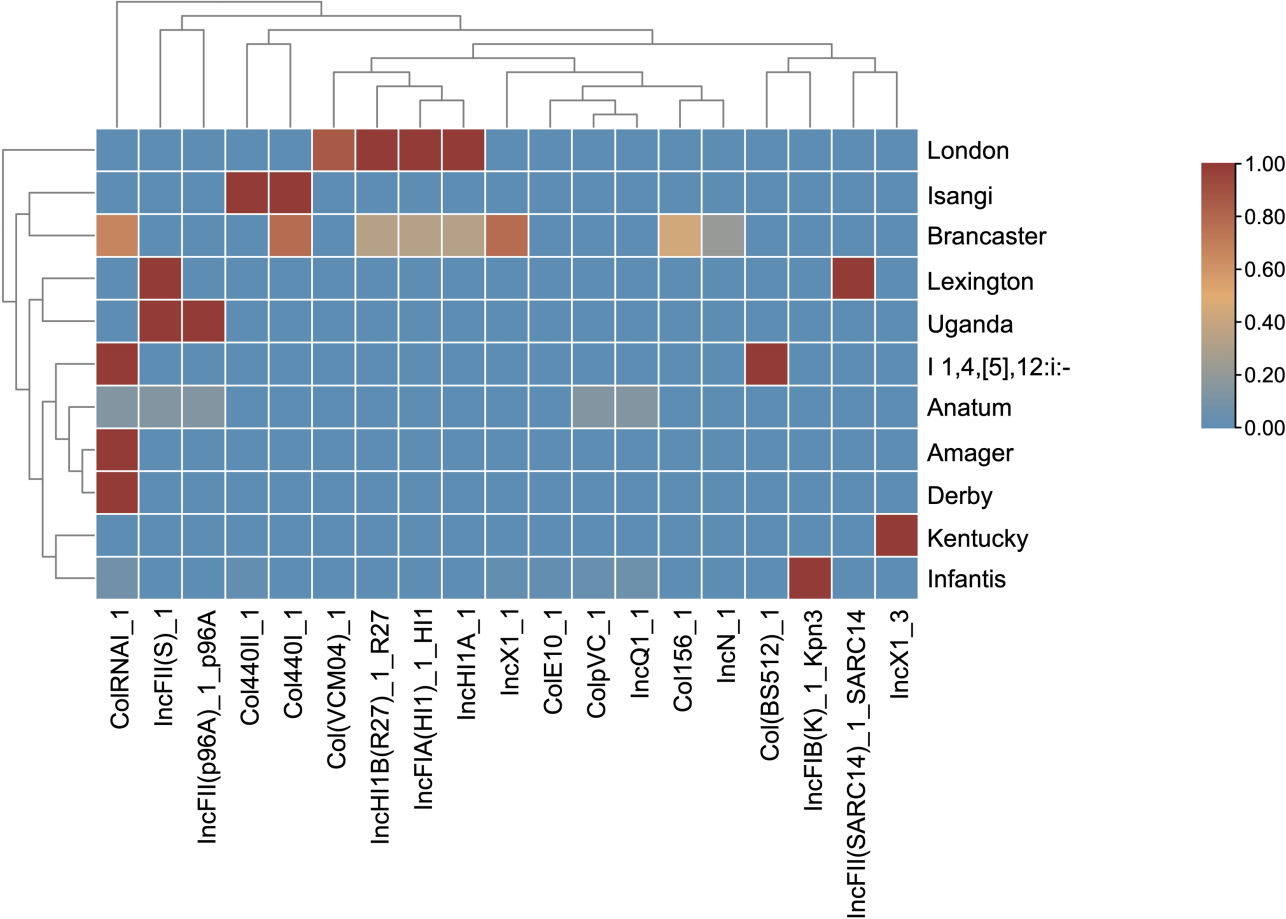
Heat map of the plasmid profiles of *S. enterica* serovars. The plasmids are clustered by their prevalence similarities. The proportion of serovars possessing plasmids ranges from 0 to 1, where absence is 0 and is blue, and presence in all isolates is 1 and is red.

*In silico* typing using PlasmidFinder identified 19 distinct plasmid replicons in 86.32% of the isolates (*n =* 82) (Figure 4). Each isolate contained between one and eight plasmid replicons, with the highest number found in two *S.* Brancaster isolates. The most common plasmid replicon was IncFIB(K)_1_Kpn3, present in 48 of 49 *S.* Infantis isolates. This was followed by the ColRNAI replicon, which was present in *S.* Derby, monophasic *S.* Typhimurium, *S.* Amager, and selected *S.* Brancaster, *S.* Infantis, and *S.* Anatum isolates.

No plasmids were detected in *S.* Livingstone, *S.* Saintpaul, *S.* Breda, and *S.* Albany isolates. Some plasmid replicons were unique to a single serovar: Col(BS512) in monophasic *S.* Typhimurium, ColE10 in one *S.* Infantis isolate, IncFIB(K)_1_Kpn3 in the majority of *S.* Infantis isolates, IncFII(SARC14) in *S.* Lexington, IncN in two *S.* Brancaster isolates, and IncX1_3 in *S.* Kentucky isolates. In contrast, other plasmid types were detected in two to five different serovars. Interestingly, IncFIA(HI1), IncHI1A, and IncHI1B(R27) were harbored by the same set of *S.* London and *S.* Brancaster isolates.

### 3.5 Virulence genes

**Figure 5:**
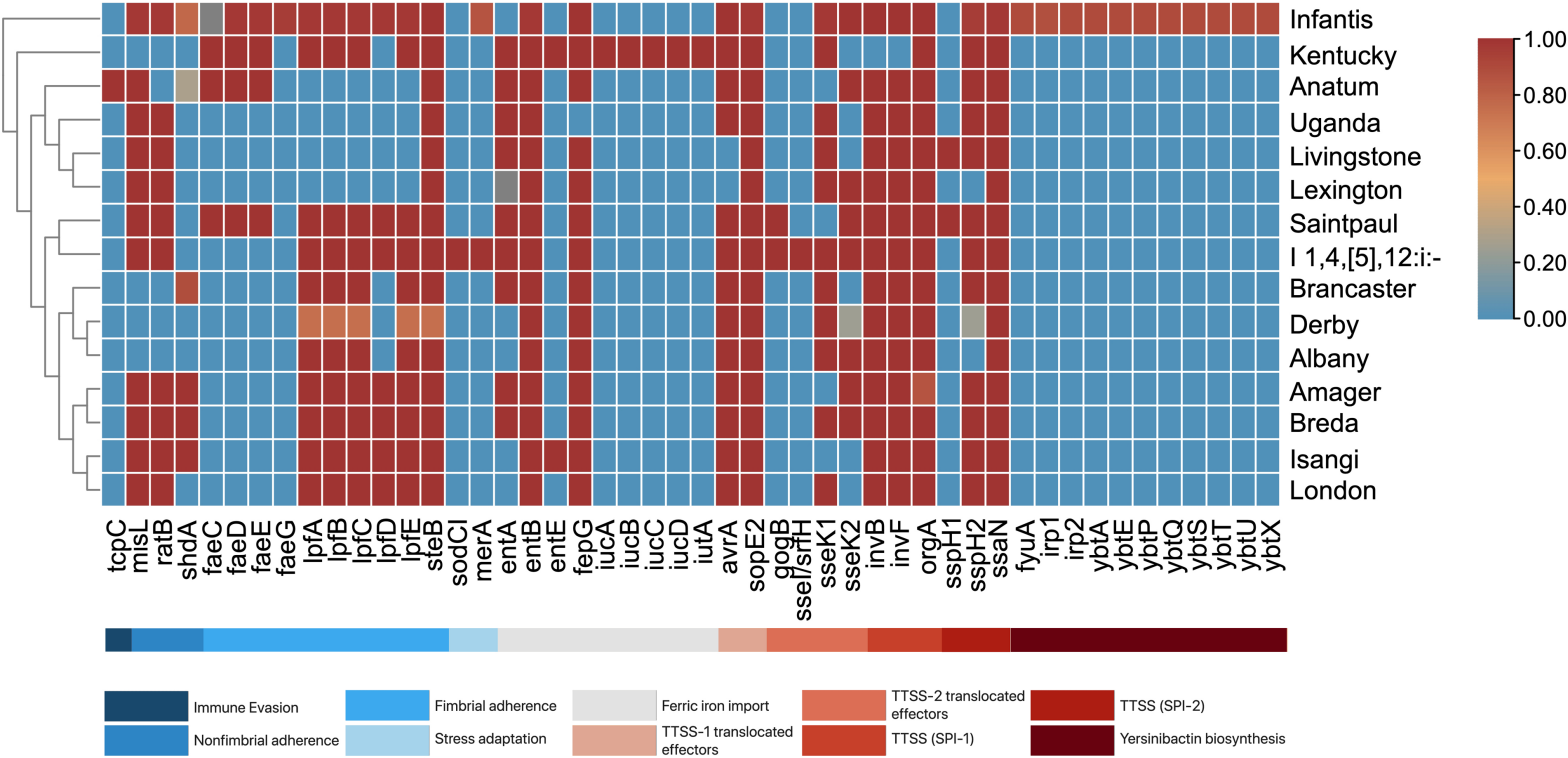
Virulence genes present among *S. enterica* serovars. The serovars are clustered by their similarities in virulence genes. The proportion of serovars possessing virulence genes ranges from 0 to 1, where absence is 0 and is blue, and presence in all isolates is 1 and is red.

A total of 131 virulence genes associated with various virulence mechanisms were identified across the *S. enterica* isolates. Each isolate contained 96–119 of these genes. Notably, 63.36% of the genes (*n =* 83) were present in all isolates, while 48 genes were identified as variable virulence factors (Figure 5).

All isolates were found to harbor genes encoding Type 1 (*fimCDFHI*) and Type 3 (*orgABC, prgHIJK, invA, sseABCDEFGm, pipB, sifAB, sipABCD, sopBDD2*) secretion systems. Among the serovars, *S.* Infantis exhibited the highest number of virulence factors (117 to 119 genes). This abundance is attributed to several genes exclusive to *S.* Infantis, including the *ybtAEPQSTUX* operon, *fyuA*, *irp-1*, *irp-2*, and *faeG*. Additionally, *S.* Infantis, along with monophasic *S.* Typhimurium, also contained the *mer* operon. Other notable serovar-specific genes include *tcpC* in *S.* Anatum, *sodCI,* and *sseI/srfH* in monophasic *S.* Typhimurium, and *iucABCD* and *iutA* in *S.* Kentucky.

## 4 Discussion

The results of the serovar prediction reveal a wide variety of serovars circulating in chicken meat sold in wet markets in Metro Manila. It showed the dominance of *S.* Infantis among the studied *Salmonella* isolates, which is consistent with previous studies in chicken meat in the U.S., Europe, and Asia (McMillan et al., 2022; Mora et al., 2024; Kim et al., 2024; Mattock et al., 2024). One possible reason for the increased detection of *S.* Infantis is the presence of plasmid of emerging *S.* Infantis or pESI, which has been detected in poultry, chicken meat, and clinical isolates across South America, Europe, Africa, and Asia (McMillan et al., 2022; Mattock et al., 2024). Other significant serovars detected in our study include Brancaster, Anatum, London, and Uganda, all of which have been previously detected in either poultry or poultry products (Xu et al., 2021; Khoo et al., 2023; Mora et al., 2024; Sodagari et al., 2023). Notably, we also detected the rare serovar Isangi, marking its first reported detection in the Philippines. Previous reports of serovar Isangi in Brazil and South Africa are linked to poultry production (dos Santos et al., 2023; Vilela et al., 2023). Interestingly, serovar Enteritidis was absent in our study, despite it being one of the dominant NTS serovars in clinical isolates in the country (Lagrada et al., 2022).

MLST analysis revealed that the isolates belong to 16 distinct STs and eBGs. Particularly, most isolates belonged to ST 32 (*S.* Infantis, eBG 31), consistent with the study of Mattock et al. (2024), where 99% (*n =* 5,205) of *S.* Infantis were classified as ST 32, and only few belong to ST 2283 and ST 2146. Other serovars belong to unique STs and eBGs, except for *S.* Derby, which was linked to STs 40 and 71. Sévellec et al. (2018) previously reported that serovar Derby is polyphyletic and can be divided into four distinct lineages, with ST 40 and 71 being associated with poultry. Although the monophasic *S.* Typhimurium isolates in our study shared a single eBG with the reference *S.* Typhimurium, they formed distinct but closely related sub-clade from the reference due to differences in sequence types (STs 4,431 and 19, respectively). Documentation on ST 4431 in literature is scarce and those that are deposited in EnteroBase are associated with human and clinical isolates. This study is among the few to report this ST in animal meat, and its previous isolations in humans might present a possible health risk.

All the serovars were isolated from a single city except for monophasic *S.* Typhimurium, *S.* Anatum, and *S.* Infantis. Serovars that were isolated from a single location and formed a single clade could be possibly sourced from the same farm or slaughterhouse. In contrast, serovars isolated from multiple locations and still formed a single clade could be attributed to farms supplying live birds to various slaughterhouses, which then distributed chicken meat from these slaughterhouses to different wet markets across cities (Mora et al., 2024).

A significant proportion of our isolates exhibited resistance to at least one antimicrobial drug class, with 45.26% categorized as MDR. Phenotypic resistance was notably high against β-lactams (penicillins, cephems, and monobactams), aminoglycosides, and nitrofurans. In serovars Uganda, Anatum, and monophasic Typhimurium, β-lactam resistance can be partly attributed to *ampH* gene and the multidrug efflux pump *mdsABC*. For serovars Infantis, London, and Brancaster, resistance is associated with β-lactamase gene variants: *bla*_CTX-M-65_, *bla*_TEM-1_, and *bla*_TEM-176_, respectively. Previous studies have reported the presence of these *bla* genes in these serovars from chicken meat (Chin et al., 2017; Brown et al., 2018; Wang et al., 2023). Particularly concerning is the *bla*_CTX-M-65_ variant in *S.* Infantis, which has facilitated its emergence and spread in poultry and its products (Alzahrani et al., 2023). Genetically related strains of *S.* Infantis carrying *bla*_CTX-M-65_ have also been found in retail meat and human isolates in the U.S. (Brown et al., 2018). The presence of ESBLs in these isolates is alarming, as it removes ceftriaxone and ampicillin as treatment option against salmonellosis (Brown et al., 2018).

Resistance to aminoglycosides, such as tobramycin (TOB, 47.37%), and gentamicin (GEN, 44.21%), was also common among the isolates. This may be due to the presence of *acrD* efflux pump or drug inactivation through aminoglycoside-modifying enzymes (AMEs). Several AME genes were detected, including variants encoding acetyltansferases (*aac(3)-IId, aac(3)-IV, aac(6′)-Iy, aac(6′)-Iaa*), nucleotidyltransferases (*ant(3″)-IIa, aadA2,* and *aadA22*) and phosphotransferases (*aph(4)-Ia,* and *aph(6)-Id*). Isolates did not show detectable resistance genes against nitrofurans (*nfsA* and *nfsB*), suggesting that resistance might be due to unknown resistance mechanisms or new ARG variants that are yet to be discovered (Alzahrani et al., 2023; Petrin et al., 2023). The use of nitrofurantoin in humans for urinary tract infections and in animals as growth promoters (Mohakud et al., 2023) may have contributed to selective pressure for new ARGs.

Resistance to ciprofloxacin (CIP, 13.68%) and trimethoprim-sulfamethoxazole (SXT, 11.58%) were less common among our isolates. In our *S.* Infantis isolates, ciprofloxacin resistance appears to be partly attributed to a point mutation in *gyrA* (D87Y), a key target for quinolones (Qian et al., 2020). Additionally, the plasmid-mediated quinolone resistance gene *qnrS1* was identified, and co-carried with *bla*_TEM-1_ *and bla*_TEM-176_ in *S.* London and *S.* Brancaster, respectively. Although *qnr* expression has been shown to reduce the bactericidal efficacy of ciprofloxacin (Chen et al., 2024), both serovars remained susceptible. This suggests that the resistance conferred by *qnrS1* in these serovars is insufficient to meet the resistance threshold for ciprofloxacin, implying that the acquisition of other *qnr* gene variants or mutations in the topoisomerase genes may be needed to achieve resistance (Salah et al., 2019). As for trimethoprim-sulfamethoxazole, the presence of both *dfrA* and *sul* gene variants in *S.* London (*dfrA12 and sul2*) and *S.* Infantis (*dfrA*14 and *sul1*) might explain their resistance. These resistance genes are linked to either class 1 integrons (*dfrA12, dfrA14,* and *sul1*) or small nonconjugative plasmids (*sul2*) (Antunes et al., 2005).

Notably, all isolates were susceptible to tigecycline (TGC), despite the presence of multidrug efflux pump *acrAB*. This may be explained by a higher epidemiological cut-off value for tigecycline compared to the resistance level conferred by the ARGs (Petrin et al., 2023), resulting in isolates being classified as susceptible. Additionally, our isolates were susceptible to both ertapenem and meropenem, and ARGs related to carbapenem resistance were not detected.

The majority of the MDR isolates (37 of 43) belonged to serovar Infantis. The proportion of MDR among Infantis isolates in our study (75.51%) is comparable to the 75% observed in South America (76%), but higher than rates reported in Asia (55%), Europe (42%), and North America (27%) (Mattock et al., 2024). This may be attributed to the ARGs possessed by the isolates, wherein each genome contained 22 to 33 ARGs, with 81.63% of the isolates harboring *bla*_CTX-M-65_. In addition, it has been found that *S.* Infantis isolates from poultry and poultry products have considerably more ARGs compared to human and environmental isolates, and 73% of poultry isolates across continents were MDR (Mattock et al., 2024). This only highlights the role of *S.* Infantis in poultry and poultry products as a major reservoir of ARGs, and emphasizes the potential risks associated with the spread of these resistance genes in humans and the environment.

The spread of ARGs can be linked to HGT facilitated by mobile genetic elements, such as plasmids. Particularly concerning are conjugative plasmids (IncC, IncF, IncHI, IncN, and IncX) that are self-transmissible, allowing them to increase the spread of ARGs (McMillan et al., 2020a; Wang and Dagan, 2024). Among the plasmids identified in our isolates, IncFIB(K)_1_Kpn3 was the most common, being identified in *S.* Infantis isolates. IncFIB(K)_1_Kpn3 is recognized as one of the contributors to the dominance of the serovar in poultry in Europe and the U.S. (Alzahrani et al., 2023), and is known to carry *bla*_CTX-M-65_ and ARG variants of *dfrA, floR, aph,* and *aac* (Hull et al., 2022; Russo et al., 2024). Other MDR plasmids identified in the study include IncFIA(HI1)_1_HI1 and IncX1_1. These plasmids are recognized for carrying a class 1 integron, as reported by several studies (Juraschek et al., 2021; Syed Abu Thahir et al., 2023; Puangseree et al., 2024). The IncFIA(HI1)_1_HI1 in our *S.* London isolates carries a diverse array of resistance genes, including *cmlA1, florR, tetA, bla*_TEM-1_*, sul2, aadA2, ant3, qnrS1,* and *dfrA12.* As for IncX1 detected in *S.* Brancaster, it carries *aph(3′)-Ia, bla*_TEM-176_, *dfrA14, floR, qnrS1,* and *tetA*, which is consistent with the findings of previous studies (Juraschek et al., 2021; Syed Abu Thahir et al., 2023).

Virulence genes play a crucial role in enabling *Salmonella* to cause disease by allowing it to survive and establish infection in the host (Retamal et al., 2022). Key virulence genes that encode Type 1 and Type 3 secretion systems, crucial for the initial stages of *Salmonella* invasion (Bao et al., 2020), were found in all isolates. Other virulence genes were exclusively found in specific serovars, enhancing their adaptive capacity to survive and cause infection (Retamal et al., 2022). In our study, we identified *sodCI* and *sseI/srfH* exclusively in serovar monophasic Typhimurium. The *sodCI* encodes a periplasmic superoxide dismutase, which protects *S.* Typhimurium from phagocytic superoxide (Tidhar et al., 2015). Meanwhile, the *sseI/srfH* gene contributes to the serovar’s ability to maintain long-term system infection in the spleen and liver (McLaughlin et al., 2009). We also found *iucABCD* and *iutA* genes exclusively in *S.* Kentucky. These genes encode for aerobactin, a siderophore that enhances the serovar’s survivability during systemic dissemination by aiding in iron acquisition (Fricke et al., 2009; Wellawa et al., 2020).

Among the *S. enterica* isolates, *S.* Infantis isolates showed the highest number of virulence determinants attributed to its serovar-specific genes. In particular, the presence of genes encoding the Ybt system (*fyuA, irp1, irp2,* and *ybtAEPQSTUX*) was detected in selected isolates. These genes encode for yersiniabactin, a siderophore that increases the ability of *Salmonella* to survive in low-iron environments (Russo et al., 2024). Since *Salmonella* growth is restricted in low-iron conditions found in eggs and live poultry, the presence of the Ybt system gives *S.* Infantis a significant advantage, enabling it to thrive where other strains struggle (McMillan et al., 2020b). Additionally, 47 of 49 *S.* Infantis isolates carried the *faeG* gene, which encodes fimbriae that enhance host colonization capability (Lee et al., 2021). Both *S.* Infantis and monophasic *S.* Typhimurium also harbored the *mer* operon, conferring resistance to mercury. Co-selection of antimicrobial and heavy metal resistance is common among Gram-negative bacteria (Mustafa et al., 2021), and the horizontal transfer of these determinants within the *S.* Infantis population constitutes a public health risk. Overall, the presence of *ybt* operon, *faeG, mer* operon, along with IncFIB(K)_1_Kpn3 and *bla*_CTX-M-65_ confers the *S.* Infantis isolates the pESI-like characteristic (García-Soto et al., 2020; Russo et al., 2024). This makes it the first documented pESI*-*like characteristics in *Salmonella* in the Philippines. These multiple resistance determinants have positioned *S.* Infantis as an emerging dominant serovar, particularly in broiler and chicken meat (Mughini-Gras et al., 2021).

The emergence of pathogenic MDR *S. enterica* strains poses significant food safety risks, underscoring the need for robust epidemiological monitoring and effective mitigation strategies across the food chain (Tang et al., 2022). The historical use of antimicrobials in livestock and poultry for disease treatment, prevention, and growth promotion has exerted selective pressure that drives the emergence of AMR. In the Philippines, poultry farms have reported the use of a range of antimicrobials, including aminoglycosides, fluoroquinolones, macrolides, penicillins, phenicols, phosphonics, polypeptides, tetracyclines, and folate pathway inhibitors (Barroga et al., 2020). At the farm level, animals are often asymptomatic carriers of *Salmonella*, which, along with their acquired ARGs, can easily spread to humans and the environment (Tang et al., 2022). Studies have shown that ARGs can contaminate air, water, and soil impacted by livestock waste (He et al., 2020). It is also possible that spread of MDR *Salmonella* happens through the meat distribution chain, such as in slaughterhouses and wet markets, where surfaces can harbor resistant bacteria, potentially stabilizing ARGs within food processing environments (Petrin et al., 2023).

Our study is part of a bigger project that aimed to recover *S. enterica* from pork and chicken samples from cities in Metro Manila. This serves as a baseline set of genomic data for *S. enterica* from chicken meat in Metro Manila, which can be used as a basis for lobbying national efforts for further research and coordinated AMR surveillance in the Philippines.

## 5 Conclusion

This study revealed a wide variety of *S. enterica* serovars in chicken meat sold in wet markets in Metro Manila, with *S.* Infantis identified as the predominant serovar. The majority of isolates exhibited resistance to at least one antimicrobial class, and some possess the MDR phenotype. The presence of plasmids that may carry multiple ARGs, along with several virulence determinants, facilitated the persistence of *Salmonella* in chicken meat, posing a major food safety concern. It is therefore imperative to limit the risk of MDR *Salmonella* spreading to humans, other animals, and the environment. With the increasing accessibility of WGS, deeper insights into the genetic basis of MDR emergence are now available. This will aid in the development of targeted control strategies to curb the spread of AMR among bacterial populations.

## Data Availability

The datasets presented in this study can be found in online repositories. The names of the repository/repositories and accession number(s) can be found in the article/Supplementary material.
